# A heterozygous *GRID2* mutation in autosomal dominant cerebellar ataxia

**DOI:** 10.1038/s41439-022-00204-x

**Published:** 2022-07-27

**Authors:** Kishin Koh, Haruo Shimazaki, Matsuo Ogawa, Yoshihisa Takiyama

**Affiliations:** 1 Department of Neurology, Graduate School of Medical Sciences, University of Yamanashi, Yamanashi, 409-3898 Japan; 2 Faculty of Health & Medical Care, Saitama Medical University, Saitama, 350-1241 Japan; 3 Mooka Chuo Clinic, Tochigi, 321-4337 Japan

**Keywords:** Spinocerebellar ataxia, Next-generation sequencing

## Abstract

A heterozygous mutation in *GRID2* that causes SCAR18 was first reported in an Algerian family with autosomal dominant cerebellar ataxia (ADCA). We identified the second ADCA family with a heterozygous *GRID2* mutation. The Algerian family had cognitive impairment and hearing loss associated with cerebellar ataxia. However, the Japanese family presented here showed pure cerebellar ataxia. Therefore, we should also screen for the *GRID2* mutation in ADCA families with pure cerebellar ataxia.

Spinocerebellar ataxias are heterogeneous disorders that include chronic progressive cerebellar ataxia. Spinocerebellar ataxias with autosomal dominant inheritance are termed SCAs, and those with autosomal recessive inheritance are termed SCARs^1,2^. Molecular genetic research has revealed many causative genes and variants for the hereditary forms of spinocerebellar ataxia. Interestingly, SCAs and SCARs have overlapping causative genes, including *SPTBN2* (SCA5/SCAR14), *GRM1* (SCA44/SCAR13), and *STUB1* (SCA48/SCAR16)^3–8^. A homozygous *GRID2* mutation was first reported to be a cause of SCAR18 in 2013^9^. In 2015, a heterozygous *GRID2* mutation was also reported as a cause of autosomal dominant cerebellar ataxia (ADCA)^10^. Here, we report the clinical and genetic features of the second ADCA family with a heterozygous *GRID2* mutation.

The family tree is shown in Fig. 1A. The affected individuals in this family were the proband (II-1), his mother (I-2), and his sisters (II-2 and II-3). The proband’s father (I-1) did not show any neurological symptoms. We collected their clinical information from medical records and from our examination.

**Fig. 1:**
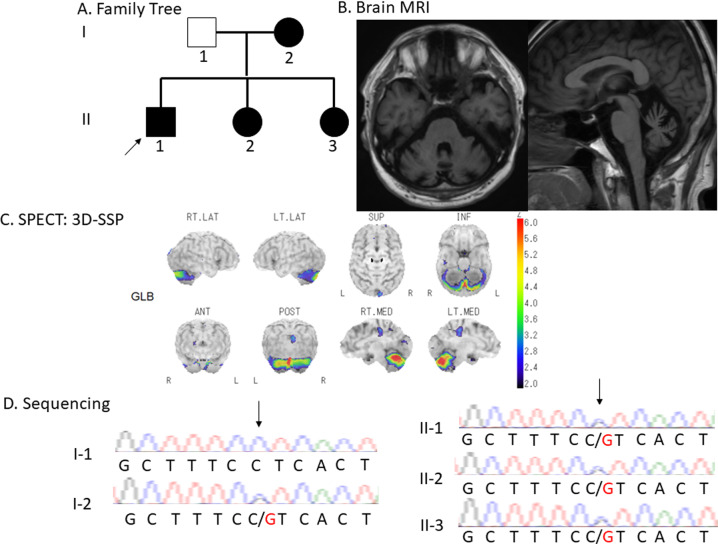
Clinical and genetic information. **A** Family tree. Squares and circles indicate males and females, respectively. Filled symbols indicate affected individuals. The arrow indicates the proband. **B** Brain MRI. Axial and sagittal slices of a T1-weighted image obtained from brain MRI in Patient II-1 showing cerebellar atrophy. **C** SPECT. 123I-IMP SPECT shows reduced cerebellar blood flow. **D** Sanger sequencing. Green, red, black, and blue curves represent adenine (A), thymine (T), guanine (G), and cytosine (C), respectively. The arrows indicate the position of the heterozygous *GRID2* mutation in the affected individuals and the unaffected one.

We also collected genomic DNA from all family members (I-1, I-2, II-1, II-2, and II-3). We first excluded pathogenic repeat expansions in SCA1 and 2, MJD/SCA3, 6, 7, 8, 17, 31, and 36, and DRPLA. Then, we conducted whole-exome sequence analysis for all participants. We screened for the known genes in SCAs (SCA5, 11, 13, 14, 15, 17, 19, 21, 22, 23, 26, 27, 28, 34, 35, 37, 38, 40, 41, 42, 43, 44, 45, 46, 47, and 48) and SCARs (SCAR1, 2, 3, 4, 5, 7, 8, 9, 10, 11, 12, 13, 14 15, 16, 17, 18, 19, 20, 21, 22, 23, 24, 25, 26, 27, 28, 29, 30, and 31). We performed Sanger sequencing to confirm the variant in all participants. Functional prediction was conducted by in silico analysis, including PolyPhen2^11^, SIFT^12^, and CADD^13^. Furthermore, we classified the variants according to ACMG guidelines^14^. This study was approved by our institutional review board, and written informed consent was obtained from all participants.

The present family included four patients with cerebellar ataxia in two generations. Their clinical information is shown in Table 1. The ages of onset in the four patients ranged from their 30s to their 50s. Symptoms at onset were gait disturbance and/or dysarthria. Their grandmother had gait instability, although we could not collect neurological information or genomic DNA for her. All patients exhibited truncal and limb ataxia without eye movement disorders, pyramidal tract signs, or sensory disturbance. Cognitive impairment and hearing loss were absent in all patients. The Scale for the Assessment and Rating of Ataxia (SARA) scores at the first examination for I-2, II-1 and II-2 (disease duration: approximately 15–20 years) were all approximately 10. Moreover, the SARA score for II-3 was 7 (disease duration: two years). The SARA scores slightly worsened to 12, 11, and 17 for I-2, II-1, and II-2 after five, seven, and seven years of disease duration, respectively. Brain MRI showed cerebellar atrophy in all patients who underwent this procedure (representative MRI in II-1, Fig. 1B). ^123^I-IMP SPECT in II-1 showed markedly reduced cerebellar blood flow (Fig. 1C).

**Table 1:** Clinical features of the present family.

	I-2	II-1	II-2	II-3	7 patients from [9]
Age at examination	75	53	50	47	32–68 (mean 44.4)
Age of onset	50 s	30 s	30 s	45	10–46 (mean 24.8)
Symptoms at onset	Gait disturbance	Gait disturbance	Gait disturbance Dysarthria	Gait disturbance	Gait instability: 4 persons Hearing loss: 1 person None: 2 persons
Total SARA score at first (age, y.o.)	10.5 (70)	10 (46)	10 (43)	–	–
Total SARA score at last (age, y.o.)	12 (75)	11 (53)	17 (50)	7 (47)	16 (45), 13.5 (50), 9 (47), 2.5 (32)
Cognitive impairment	–	–	–	–	3/7
Hearing loss	–	–	–	–	3/7
Cerebral MRI	NA	Cerebellar atrophy	NA	Cerebellar atrophy	Normal: 2 persons Cerebellar atrophy: 1 person Vermian atrophy: 1 person NA: 3 persons

*NA* not available.

There were no pathogenic repeat expansions in SCA1 or 2 or MJD/SCA3, 6, 7, 8, 17, or 31. Through gene screening by whole-exome sequencing, we found a heterozygous *GRID2* variant (NM_001510.4: c.1966C>G, p.Leu656Val) in all patients in this family. The *GRID2* variant allowed segregation of the affected individuals from a normal family member (I-1), as confirmed by Sanger sequencing (representative data for II-1 and I-1, Fig. 1D). The results of in silico analysis indicated this variant was probably damaging with Polyphen2, damaging with SIFT, and 27.7 with CADD. The amino acid at this position was conserved across species. According to the ACMG guidelines, the variant (NM_001510.4: c.1966C>G, p.Leu656Val) was classified as “pathogenic”.

We described the second ADCA family with a heterozygous *GRID2* mutation in the present study. To date, a heterozygous *GRID2* variant has only been described in sporadic cases outside the first family^10^. Interestingly, our family had the same mutation of *GRID2* as that in the first family^10^. Therefore, we could state that our family was the second family with a heterozygous p.Leu656Val *GRID2* mutation. *GRID2* encodes the GluRD2 protein, which generally comprises extracellular domains (S1 and S2), transmembrane segments (M1, M3, and M4), and linkers (S1M1, M3S2, and S2M4)^15,16^. The p.Leu656Val mutation in the two families existed in the M3S2 linker. Furthermore, according to an earlier report^10^, three missense mutations (p.Ala654Thr, p.Ala654Asp, and p.Leu656Val) near the M3S2 linker cause cerebellar ataxia. Additionally, seven-point variants (p.Trp18Arg, p.Thr282Met, p.Arg304*, p.Thr330Met, p.Ala654Thr, p.Ala654Asp, and p.Leu656Val) have been reported to be heterozygous *GRID2* variants in five reports to date (Table 2)^10,17–20^. Heterozygous missense mutations could lead to a GRID2 loss of function, which would be expected due to the recessive inheritance pattern. Three missense mutations (p. Ala654Thr, p.Ala654Asp, and p.Leu656Val) and one nonsense variant (p.Arg304*) were not registered in gnomAD^21^. Moreover, three missense variants (p.Trp18Arg, p.Thr282Met, and p.Thr330Met) were registered in gnomAD. According to these results and the ACMG guidelines, p.Trg18Arg, p.Thr282Met, p.Arg304*, and p.Thr330Met are classified as “likely benign”, “uncertain significance”, “pathogenic”, and “likely benign”.

**Table 2:** Reported heterozygous and homozygous variants in *GRID2*.

Variation	Trp18Arg	Thr282Met	Arg304*	Thr330Met	Ala654Thr	Ala656Asp	Leu656Val	Leu656Val
Zygosity	Hetero	Hetero	Hetero	Hetero	Hetero	Hetero	Hetero	Homo
Family history	–	–	–	–	–	–	+	+
gnomAD	+	+	–	+	–	–	–	–
ACMG criteria	Likely benign	Uncertain significance	Pathogenic	Likely benign	Pathogenic	Pathogenic	Pathogenic	Pathogenic

Thus, the *GRID2* mutation of p.Leu656Val would be considered pathogenic. However, the causes of the differences in severity, including ataxia, cognitive impairment, and hearing loss, between the first family and the present remain unknown. The present family exhibited only cerebellar ataxia, which indicated a phenotype of pure cerebellar ataxia. However, some patients in the first ADCA family showed cognitive impairment and hearing loss, indicating intrafamilial clinical variability (Table 1). We only know that the disease duration and severity change in parallel.

In summary, we identified the second ADCA family with the heterozygous mutation (NM001510.4: c.1966C>G, p.Leu656Val) in the *GRID2* gene; this variant was found in an Algerian family using whole-exome analysis. We should screen for *GRID2* variants in the case of families with pure cerebellar ataxia in ADCA. Further studies are required to elucidate the genotype-phenotype correlation in *GRID2*-related ataxias.

## Data Availability

The relevant data from this Data Report are hosted at the Human Genome Variation Database at 10.6084/m9.figshare.hgv.3207.
